# A new species of *Heser* Tuneva, 2005 (Araneae, Gnaphosidae) from the south of India

**DOI:** 10.3897/zookeys.73.837

**Published:** 2010-12-29

**Authors:** Jan Bosselaers

**Affiliations:** Section of invertebrates, Royal Museum for Central Africa, B-3080 Tervuren, Belgium

**Keywords:** Dionycha, Karnataka, Hampi, Zelotinae, key, *Aponedyopus*

## Abstract

A new species of Heser Tuneva, 2005 (Gnaphosidae) is described from the south of India. A key is provided to the species of Heser and the importance of Gnaphosidae for the study of world spider biodiversity is briefly discussed.

## Introduction

The genus Heser Tuneva, 2005 belongs in the gnaphosid subfamily Zelotinae by the presence of metatarsal preening combs on legs III and IV, and was delimited by Tuneva (2005) based on the possession of a male palp with an elongated, hook-shaped median apophysis, and with a prolateral-basally inserted embolus which has a relatively narrow base and which is extending transversally across the distal end of the palpal bulb. Moreover, the genus can be distinguished from Zelotes Gistel, 1848 by lacking an intercalary sclerite and terminal apophysis of the male palp, as well as by the absence of coiled median and blind paramedian vulval ducts and the presence of glands on the insemination ducts in females, and it differs from most other zelotine genera by its large posterior median eyes. As a consequence, Heser resembles Camillina Berland, 1919 by the shape of the posterior median eyes, wich are large, subtriangular and almost contiguous. However, it differs from that genus by the absence of a bifid, prolateral palpal terminal apophysis, by a different shape of the embolar base and by the absence of a subtriangular to hemicircular anterior median epigynal plate. From Drasyllus Chamberlin, 1922, Heser also differs by the absence of a bifid palpal terminal apophysis or a median epigynal plate, while it can be easily distinguished from Setaphis Simon, 1893 by the absence of a terminal coil on the embolus and from Zelowan Murphy & Russell-Smith, 2010 by its retrolateral tibial apophysis, which does not consist of two small triangular lobes. At present, Heser counts three species: the type species Heser malefactor Tuneva, 2005, as well as Heser aradensis (Levy, 1998) and Heser infumatus (O. Pickard-Cambridge, 1872) which were transferred from Zelotes by Tuneva (2005). The genus has a known distribution area in the eastern Mediterranean, tropical Africa and central Asia (Murphy 2007). On an archaeological mission to the south of India in 2006 (Bosselaers and Valcke 2009), the author collected both sexes of a new species of Heser among the ruins of Hampi, the former capital of the Vijayanagar empire (Filliozat 2004, Fritz and Michell 2001, Verghese 2002). The new species expands the range of the genus to southern India (Fig. 20).

## Material and methods

The specimens were observed, photographed and drawn under Euromex MIC 465 and Olympus SZC-X9 binocular microscopes equipped with an eyepiece grid and a Praktica DC42 digital camera.. The vulva (cleared in methyl salicylate) was observed and drawn using a Wild M12 compound microscope. All measurements are in mm, unless otherwise stated. The format for leg spination follows Platnick and Shadab (1975), amended for ventral spine pairs according to Bosselaers and Jocqué (2000). Leg spination is also illustrated in a schematic representation (Figs 9, 11) where pl, do, rl and ve sides of leg articles are flattened as a folding net (Dürer 1525, Bosselaers 2009).

### Abbreviations

AERanterior eye row; ALEanterior lateral eyes; ALS anterior lateral spinnerets; AMEanterior median eyes; fefemur; MOQmedian ocular quadrangle; mtmetatarsus; papatella; PERposterior eye row; PLEposterior lateral eyes; PLSposterior lateral spinnerets; PMSposterior median spinnerets; titibia.

### Abbreviation of institutional collection (curator in parentheses):

RBINSRoyal Belgian Institute of Natural Sciences, Brussels (L. Baert)

## Taxonomy

**Heser Tuneva, 2005**

### Heser vijayanagara sp. n.

urn:lsid:zoobank.org:act:4CD46D69-8560-49BD-B58F-825F96510C14

Figs 1–8, 10–11 12–19 

#### Type material.

Holotype male:India, Karnataka, Hospet, Hampi World Heritage Site, 15°18'27.7"N 76°28'32.8"E, alt. 455 m, under stone close to archaeological office, 25 November 2006, J. Bosselaers leg. (RBINS). Allotype female, same data (RBINS).

#### Diagnosis.

The species can be distinguished from the other three species of Heser by the abdominal dorsal scutum in males, the pronounced S-shaped curve of the sperm duct, the small, transversally oriented median apophysis and the hook-shaped embolus tip circling the broad membranous conductor in the male palp, as well as the narrow anterior epigynal hood, the large spermathecae and the circular insemination ducts in females.

#### Entymology.

The species epithet is a noun in apposition and refers to the imperial city of Vijayanagara (Hampi, Karnataka, India) among whose ruins the type specimens of the new species were found (Fig. 9).

#### Description.

##### Male (holotype).

**Figures 1–11.**
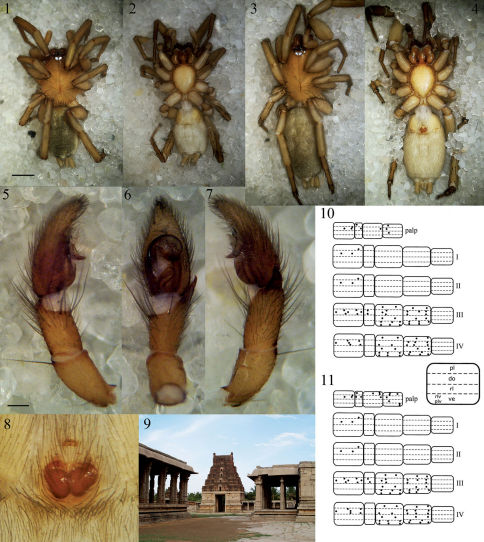
Heser vijayanagara sp. n. **1** Male holotype, dorsal **2** Male holotype, ventral **3** Female allotype, dorsal **4** Female allotype, ventral **5** Male palp, prolateral **6** Male palp, ventral **7** Male palp, retrolateral **8** Epigyne, ventral **9** Pattabhirama temple in close vicinity of the *locus typicus*, giving a good impression of the type of terrain where the type specimens were found **10** Male leg spination diagram, legend below right **11** Female leg spination diagram. Scale bars: 1–4: 1.0; 5–8: 0.25.

Total length: 5.00. Carapace length: 2.24; width: 1.63. Carapace orange brown, unicolorous, with a deep and narrow fovea in the posterior half (Fig. 1). Eight eyes in two rows, ringed with black, AER width 0.47, straight from above, slightly procurved from front, PER width 0.56, procurved from above, strongly procurved from front. MOQ depth 0.35, anterior width 0.29, posterior width 0.32. Eyes of AER subequal, AME grey and circular, separated by half their diameter, ALE oval, pearly white, touching AME. PME oval to subtriangular, pearly white, touching, larger than AME (Fig. 1). PLE subquadratic, pearly white, slightly smaller than ALE, separated from PME by half the PLE diameter. Clypeus vertical, slightly larger than diameter of AME. Chilum small, sclerotised, subtriangular and single, orange-brown. Chelicerae brown, with a few scattered thin setae on anterior face, anterior cheliceral rim with two very small teeth close to fang base and three larger teeth further from fang base, posterior cheliceral rim with one very small tooth close to fang base end two medium-sized teeth further from fang base. Sternum smooth, orange-brown, shield-shaped with a thin border, length 1.32; width 1.08. No precoxal triangles (Bosselaers and Jocqué 2002: 247, fig. 1K, Penniman 1985: 16) or intercoxal sclerites (Bosselaers and Jocqué 2002: 247), pleural bars (Bosselaers and Jocqué 2002: 247, fig. 1P; Simon 1892: 11, fig. 29) yellow-brown, weakly sclerotised and isolated, not protruding between coxae. Labium brown, longer than wide, with a slightly thickened anterior rim. Endites longer than labium, dumbbell-shaped with oblique depression, and provided with an apical hair tuft (Fig. 2). Abdomen mottled grey dorsally, with a frontal row of curved hairs and a small, shiny, brown anterior do scutum covering less than 10% of abdominal do surface area. (Fig. 1). Lateral and ventral sides of abdomen creamy white (Figs 1, 2). ALS and PLS large and cylindrical, PMS thin and slender, shorter than PLS. Legs yellow-brown, unicolorous (Figs 1, 2). No trochanter notch, no retrocoxal hymen (Raven 1998, Bosselaers and Jocqué 2002), patellar indentation (Simon 1892: 22, Ledoux and Canard 1991: fig. 15A-15B) long and narrow, 2/3 of pa length. Metatarsi III and IV with ventral terminal preening comb composed of stiff, black setae. Tarsi with two pectinate claws, no claw tufts. Leg formula 4123. Leg spination (Fig. 10): fe: palp do 0–1-2; I pl 0–0-1 do 1–1-0;II pl 0–0-1 do 1–1-0;III do 1–3-3; IV do 1–3-2; pa: palp pl 1–0 do 0–1; III do 0–2-0; ti: palp do 0–0-1; III pl 1–0-1 do 3–0-0 rl 1–0-1 ve 2–2-2; IV pl 1–0-1 do 1–1-0 rl 1–0-1 ve 2–2-2; mt: III pl 0–1-1 do 2–1-2 rl 0–1-1 ve 2–2-1; IV pl 0–1-1 do 2–2-2 rl 0–1-1 ve 2–2-1; ta: palp pl 1–0-0 do 1–0-0 rl 1–0-0.

**Figures 12–19.**
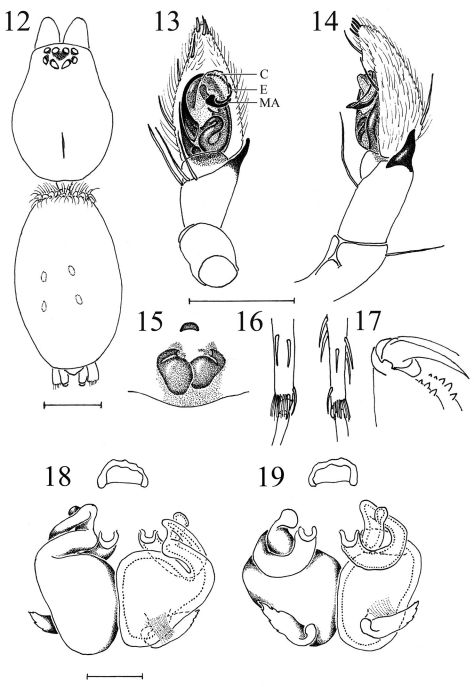
Heser vijayanagara sp. n. **12** Female allotype, dorsal view of body **13** Male palp, ventral, with conductor (C), embolus (E), and median apophysis (MA) indicated **14** Male palp, retrolateral **15** Epigyne, ventral **16** Female metatarsi III (left) and IV, with ventral terminal preening comb **17** Female cheliceral teeth **18** Vulva, ventral **19** Vulva, dorsal. Scale bars: 12: 1.0; 13–17: 0.5; 18–19: 0.1.

###### Leg measurements:

**Table:** 

	fe	pa	ti	mt	ta	Total
I	1.66	0.97	1.45	1.21	0.92	6.21
II	1.34	0.74	1.08	1.08	0.87	5.10
III	1.16	0.63	0.89	1.03	0.74	4.44
IV	1.71	0.87	1.42	1.66	1.03	6.68

Male palp with a slender, basally-prolaterally inserted embolus circling more than half of the tegulum, having a hook-shaped tip pointing in prolateral direction, which is curling around the broad, membranous conductor. Median apophysis small and subtriangular, oriented transversally. Sperm duct with a pronounced, S-shaped curve in basal half of tegulum. Retrolateral tibial apophysis pointed, subtriangular (Figs 5–7, 13–14).

##### Female (allotype).

Total length: 6.31. Carapace length: 2.37; width: 1.79. Carapace as in male (Fig. 3). Eyes as in male, AER width 0.53, PER width 0.58, MOQ depth 0.39, anterior width 0.30, posterior width 0.31. PME subtriangular, somewhat smaller than in male, almost touching (Figs 3, 12). Clypeus and chilum as in male. Cheliceral teeth as in male (Fig. 17). Sternum smooth, yellow-brown with a darker margin (Fig. 4), shield-shaped with a thin border, length 1.45; width 1.13. No precoxal triangles or intercoxal sclerites, pleural bars as in male. Labium and endites as in male (Fig. 4). Abdomen pale grey dorsally, with a frontal row of curved hairs and a number of paler chevrons in posterior half, no do scutum (Fig. 3). Ventral side of abdomen pale white (Fig. 4). Legs yellow-brown, unicolorous (Figs 3, 4). No trochanter notch, no retrocoxal hymen, patellar indentation as in male. Metatarsi III and IV with ventral terminal preening comb composed of stiff, black setae (Fig. 16). Tarsi with two pectinate claws, no claw tufts. Leg formula 4123. Leg spination (Fig. 11): fe: palp do 0–1-2; I pl 0–0-1 do 1–1-0;II pl 0–0-1 do 1–1-0;III do 1–3-3; IV do 1–3-2; pa: palp pl 1–0 do 1–1; III do 0–2-0; ti: palp pl 0–1-2 do 0–0-1; III pl 1–0-1 do 3–0-0 rl 1–0-1 ve 2–2-2; IV pl 1–0-1 do 1–1-0 rl 1–0-1 ve 2–2-2; mt: III pl 0–1-1 do 2–1-2 rl 0–1-1 ve 2–2-1; IV pl 0–1-1 do 2–2-2 rl 0–1-1 ve 2–2-1; ta: palp pl 0–2-1 do 1–0-0 rl 0–1-0 ve 0–0-2.

###### Leg measurements:

**Table:** 

	fe	pa	ti	mt	ta	Total
I	1.63	0.92	1.34	1.05	0.84	5.79
II	1.37	0.79	1.00	0.87	0.74	4.76
III	1.24	0.55	0.82	0.92	0.71	4.23
IV	1.71	0.84	1.34	1.58	0.92	6.39

Epigyne simple and poorly sclerotised, with a narrow anterior hood and showing large, oval spermathecae, as well as the connected, stout, inward directed part of the looped anterior insemination ducts. Copulatory openings small and medially situated (Figs 8, 15, 18, 19). Vulva (Figs 18, 19) with two large, thick-walled, oval, posterior spermathecae, connected to the anterior, medially situated copulatory openings by an insemination duct looped over 360°. The first, hemicircular stretch of the looped insemination duct passes dorsally behind the anterior part of the spermathecae, while the second, straight, longitudinally directed stretch carries an accessory gland (Fig. 19), and the third, stout, outward directed and ventrally situated stretch connects to the large spermathecae (Fig. 18).

**Figure 20.**
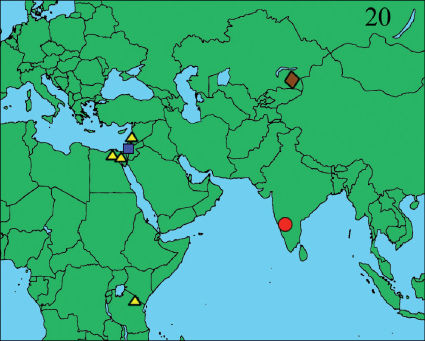
Distribution map of the four species of the genus Heser. Heser aradensis: blue square; Heser infumatus: yellow triangle; Heser malefactor: brown lozenge; Heser vijayanagara sp. n.; red circle.

## Key to the species of Heser

**Table:** 

1	Males	2
–	Females	5
2	Total length less than 4 mm. Cymbium blunt-tipped, male palpal ti longer than wide, retrolateral tibial apophysis short and blunt (Levy 1998, fig. 116)	Heser aradensis
–	Total length more than 4 mm. Cymbium pointed, male palpal ti at least as long as wide, retrolateral tibial apophysis triangular and pointed	3
3	Membranous conductor of the male palp subtriangular and more or less pointed (Levy 1998, fig. 112)	Heser infumatus
–	Membranous conductor broad and blunt	4
4	Embolus tip pointing towards bulbus base, median apophysis large, with a long basally oriented tip bent outwards (Tuneva 2005, fig. 10). No dorsal abdominal scutum	Heser malefactor
–	Embolus tip curled around conductor, pointing in prolateral direction. Median apophysis small, triangular, directed transversally (Fig. 13). Small anterior dorsal abdominal scutum.	Heser vijayanagara sp. n.
5	Total length 4 mm or less	6
–	Total length 5 mm or more	7
6	No anterior epigynal hood present, copulatory openings relatively small, their long axis directed transversally (Levy 1998, fig. 118)	Heser aradensis
–	Broad anterior epigynal hood present, copulatory openings large, their long axis oriented longitudinally (Tuneva 2005, fig. 13)	Heser malefactor
7	Anterior epigynal hood broad, copulatory openings a transversal slit, spermatheca diameter smaller than half the longitudinal dimension of the epigyne (Levy 1998, fig. 114)	Heser infumatus
–	Anterior epigynal hood narrow, copulatory openings a small oval, their long axis longitudinally oriented, spermatheca diameter about half the longitudinal dimension of the epigyne (Figs 8, 15, 18, 19)	Heser vijayanagara sp. n.

## Discussion

The family Gnaphosidae is one of the largest spider families. In Platnick (2010), it is listed as the sixth largest family, with 114 genera and 2102 species. Moreover, since Araneidae and Thomisidae, which presently count more known species, have already been studied rather intensely, also in tropical regions, it is quite probable that Gnaphosidae will prove to be the fourth largest spider family known when it has been more thoroughly revised. Indeed, in spite of a number of excellent revisions being available (Platnick and Shadab 1983, Platnick and Murphy 1984), a large number of species is still awaiting description, even in well studied regions such as Spain (Melic 2004, pers. comm.). Many newly discovered species turn out to be endemisms with relatively small distribution areas, again suggesting that many more remain to be discovered (Melic and Barriga 2007, Snazell and Murphy 1997). Revisions from tropical or subtropical gnaphosid genera regularly result in a considerable number of new species, especially in the subfamily Zelotinae, the gnaphosids with metatarsal preening combs listed by Murphy (2007) as the “Zelotes-group” (Murphy and Russell-Smith 2010, Nzigidahera and Jocqué 2009, Platnick and Murphy 1996).

The Gnaphosidae of the Indian subcontinent and its surroundings have been studied by Simon (1897), Reimoser (1934), Denis (1958) and Roewer (1961), and our knowledge about them has been compiled by Tikader (1982). Additional data were later published by (Gajbe (1987, 2005, 2007) and Butt and Beg (2004). Heser has not yet been mentioned for the region, and none of the zelotine species illustrated by the aforementioned authors resembles the genus. It is possible that Heser species are rare, as three out of the four known species are only known from the type locality. Alternatively, Heser species may be frequently overlooked because of their small size and nocturnal habits (Levy 1998).

The recent progress made in the taxonomic study of ground spiders demonstrates that, for a long time to come, Gnaphosidae, and Gnaphosoidea in general, will remain a prime target for biodiversity studies within Araneae.

## Supplementary Material

XML Treatment for Heser vijayanagara
